# α-Poly-l-lysine functions as an adipogenic inducer in 3T3-L1 preadipocytes

**DOI:** 10.1007/s00726-020-02932-2

**Published:** 2021-03-20

**Authors:** Kyeong Won Lee, Young Jun An, Janet Lee, Jung-Hyun Lee, Hyung-Soon Yim

**Affiliations:** 1 Marine Biotechnology Research Center, Korea Institute of Ocean Science and Technology, 385 Haeyang-ro, Busan, 49111 Republic of Korea; 2 Department of Ocean Science, Korea University of Science and Technology, Daejeon, 34113 Republic of Korea

**Keywords:** α-Poly-l-lysine, Adipogenesis, Insulin signaling, 3T3-L1

## Abstract

α-Poly-l-lysine (PLL) has been used for various purposes such as cell attachment, immunization, and molecular delivery, and is known to be cytotoxic to several cell lines. Here, we studied the effect of PLL on the adipogenesis of 3T3-L1 cells and investigated the underlying mechanism. Differentiation media containing PLL with a molecular weight (MW) greater than 4 kDa enhanced lipid droplet formation and increased adipogenic marker levels, indicating an increase in adipocyte differentiation. PLL with a molecular weight between 30 and 70 kDa was more effective than PLL of other sizes in 3T3-L1 cell differentiation. Moreover, PLL induced 3T3-L1 adipogenesis in insulin-free adipocyte differentiation medium. Incubation with insulin and PLL exhibited greater adipogenesis than insulin treatment only even at a high concentration. PLL stimulated insulin signaling and augmented the signaling pathway when it was added with insulin. While PLL did not activate the glucocorticoid receptor, which is phosphorylated by dexamethasone (DEX), it showed a positive effect on the cAMP signal pathway when preadipocytes were treated with PLL and 3-isobutyl-1-methylxanthine (IBMX). Consistent with these results, incubation with PLL and DEX without IBMX induced adipocyte differentiation. We also observed that the mitotic clonal expansion phase was the critical stage in adipogenesis for inducing the effects of PLL. These results suggest that PLL functions as an adipogenic inducer in 3T3-L1 preadipocytes and PLL has a direct effect on insulin signaling, one of the main regulatory pathways.

**Supplementary Information:**

The online version contains supplementary material available at 10.1007/s00726-020-02932-2.

## Introduction

Adipose tissue is a crucial regulator of glucose homeostasis and calorie storage for regulating energy balance (Rosen and Spiegelman 2006), with its dysfunction provoking metabolic diseases such as type 2 diabetes, hypertension, hyperlipidemia, and arteriosclerosis (Haslam and James 2005). Thus, knowledge of adipocyte biology is important for understanding the pathophysiological basis of obesity and metabolic diseases. 3T3-L1 cell is one of the most well-established model cell lines for studying adipocyte biology in vitro (Green and Kehinde 1975). Therefore, factors that enhance or inhibit 3T3-L1 preadipocyte differentiation have been investigated to elucidate their differentiation mechanism (Sarjeant and Stephens 2012).

Poly-l-lysine is a homopolypeptide composed of l-lysine that can polymerize via its α- (PLL) and ε-carbon (EPL). EPL is a natural product of bacterial fermentation with various known functions, including as a preservative and surfactant (Hiraki et al. 2003). α-Polylysine can consist of l- or d-lysine. PLL has been used for cell attachment (Ham and McKeehan 1979; Mazia et al. 1975), as an intermediary carrier for immunization (Manabe et al. 1986), and for the delivery of molecules such as DNA, RNA, proteins, and nanoparticles (Lemaitre et al. 1987; Jiang et al. 2016, 2015; Kadlecova et al. 2013; Park et al. 2006). Several enzymes have also been reported to be activated or inhibited by PLL (Brunati et al. 1985; Bazzi and Nelsestuen 1987; Gatica et al. 1987; Pelech and Cohen 1985). Moreover, high concentrations of PLL have cytotoxic effects on L929 mouse fibroblasts (Fischer et al. 2003), while it can improve the proliferation of preadipocytes and neuroblastoma cells in culture (Ho Kwon 2001; Bottenstein and Sato 1980). However, the effect of PLL on adipogenesis has not yet been examined, although free amino acids such as hydroxyproline, proline, lysine, glycine, and alanine induced adipogenic effects in retinal pericytes (Vidhya et al. 2018) and the catabolism of branched-chain amino acids contributed to adipocyte differentiation (Green et al. 2016). Since the positive charges of PLL enable attachment of PLL on a variety of biological molecules, PLL can be adopted as useful materials in a lot of experimental fields. However, the unknown effect of PLL may lead to unexplainable results. We also added PLL into plasmid transfection media of 3T3-L1 to increase efficiency (data not shown) and found that PLL exerts a physiological impact on 3T3-L1 cell itself. Therefore, it is important to assess the effect of PLL on 3T3-L1 adipogenesis and to understand the function of PLL when it is used as additives, mediator, and so on.

In this study, we investigated the effect of PLL on the differentiation of 3T3-L1 preadipocytes and the mechanism by which PLL contributes to adipogenesis. We found that PLL promoted 3T3-L1 preadipocyte differentiation and substituted for insulin, which is an essential component in traditional 3T3-L1 differentiation media. To elucidate how PLL affects adipogenesis, we examined the activation of the signaling pathway that is involved in the process. On the basis of our observation, we suggest that PLL activates the insulin signaling pathway and can replace insulin in 3T3-L1 adipogenic differentiation.

## Materials and methods

### Cell culture

3T3-L1 preadipocytes were maintained in Dulbecco’s modified Eagle’s medium (DMEM) supplemented with 10% bovine serum and 1% penicillin/streptomycin at 37 °C in a humidified 5% CO_2_ incubator and allowed to reach confluence (GIBCO, Life Technologies Ltd., Paisley, UK). After 2 days (Day 0), the cells were incubated in DMEM containing 10% fetal bovine serum (FBS), 0.5 mM IBMX, 0.25 μM DEX, and 5 μg/mL insulin for 2 days, and then the medium was changed to DMEM containing 10% FBS and 1 μg/mL insulin. After 2 days, the cells were maintained in DMEM containing 10% FBS for 4 days. To examine whether PLL (P0879, P6516, P9155, P1274; Sigma-Aldrich, St. Louis, MO, USA) affect adipocyte differentiation, the cells were treated with PLL or PDL throughout adipogenesis. To observe the differences in adipocyte differentiation, we used old passage cells (> passage 14) since it is difficult to monitor the effects of PLL on adipogenesis in early passage cells that have a strong differentiation potential.

#### Oil Red O staining and quantification

The cells were fixed with 3.5% formaldehyde solution for 10 min, rinsed twice with phosphate-buffered saline (PBS), and incubated with 1.5% Oil Red O in 60% isopropanol (O0625; Sigma-Aldrich) for 1 h. The cells were washed three times with PBS and dried to observe lipid droplets. For quantification of differentiated cells, stained Oil Red O was dissolved in dimethyl sulfoxide and its absorbance was measured at 510 nm.

#### Western blot analysis

Cells were lysed with lysis buffer containing 20 mM Tris–HCl (pH 7.4), 5 mM EDTA, 1% NP-40, 10 mM Na_4_P_2_O_7_, 100 mM NaF, 2 mM Na_3_VO_4_, and a protease inhibitor cocktail (#862209, ThermoFisher Scientific, MA, USA). Whole-cell lysates (10 µg) were subject to SDS-PAGE and blotted with specific antibodies. Antibodies against peroxisome proliferator-activated receptor γ (PPARγ) (sc-7273, Santa Cruz Biotech, Santa Cruz, CA, USA), CCAAT/enhancer-binding protein α (C/EBPα) (sc-61, Santa Cruz Biotech), γ-tubulin (T6557, Sigma-Aldrich), pIR (#3023, Cell signaling Technology, Beverly, MA, USA), IR (#3025, Cell signaling Technology), pIGF1R (ab39398, Abcam, Cambridge, MA, USA), IGF1R (#9750, Cell signaling Technology), pIRS1 (I2658-1VL, Sigma-Aldrich), IRS1 (#3407, Cell signaling Technology), pAkt (#4060, Cell signaling Technology), Akt (sc-8312, Santa Cruz Biotech), pERK (#4370, Cell signaling Technology), ERK (#4696, Cell signaling Technology), pGR (#4161, Cell signaling Technology), GR (#12041, Cell signaling Technology), pCREB (#9198, Cell signaling Technology), and CREB (#9197, Cell signaling Technology) were used. Blots were developed using Clarity™ Western ECL Blotting Substrates (BioRad) or ECL-Prime (ThermoFisher Scientific) with a ChemiDoc Imaging System (BioRad).

#### Real-time RT-PCR

Total RNA was extracted using TRIzol (Invitrogen, CA, USA) according to the manufacturer’s protocol and used (1 µg) to synthesize complementary DNA (cDNA) with Moloney Murine Leukemia Virus (MMLV) reverse transcriptase and random hexamers (Invitrogen). The levels of each gene transcript were assessed by real-time PCR by using gene-specific primers (Table 1) and 18S rRNA was used for normalization as an endogenous control. Real-time PCR was performed on a StepOnePlus Real-Time PCR System (Applied Biosystems) using Thunderbird SYBR qPCR Mix (TOYOBO, Japan).

**Table 1:** Primers used for real-time RT/PCR

Primers		Sequence	Species
PPARγ	F	5′-TCATGACCAGGGAGTTCCTC-3’	Mouse
	R	5′-GGCGGTCTCCACTGAGAATA-3’	
C/EBPα	F	5′-AGTCGGTGGACAAGAACAGC-3’	
	R	5′-GTCACTGGTCAACTCCAGCA-3’	
Adiponection	F	5′-GGAACTTGTGCAGGTTGGAT-3’	
	R	5′-TCTCTCCAGGAGTGCCATCT-3’	
FABP4	F	5′-AAGAAGTGGGAGTGGGCTTT-3’	
	R	5′-CTCTCTGACCGGATGGTGAC-3’	
18s rRNA	F	5′-CGCGGTTCTATTTTGTTGGT-3’	
	R	5′-AGTCGGCATCGTTTATGGTC-3’	

*F* forward, *R* reverse

### Statistical analysis

Data are presented as the average ± standard error. Statistical significance was analyzed using the Mann–Whitney *U* test or one-way ANOVA followed by Tukey’s post hoc test. Values of *P* < 0.05 were considered statistically significant.

## Results

### PLL with a molecular weight greater than 4 kDa promotes adipocyte differentiation

**Fig. 1:**
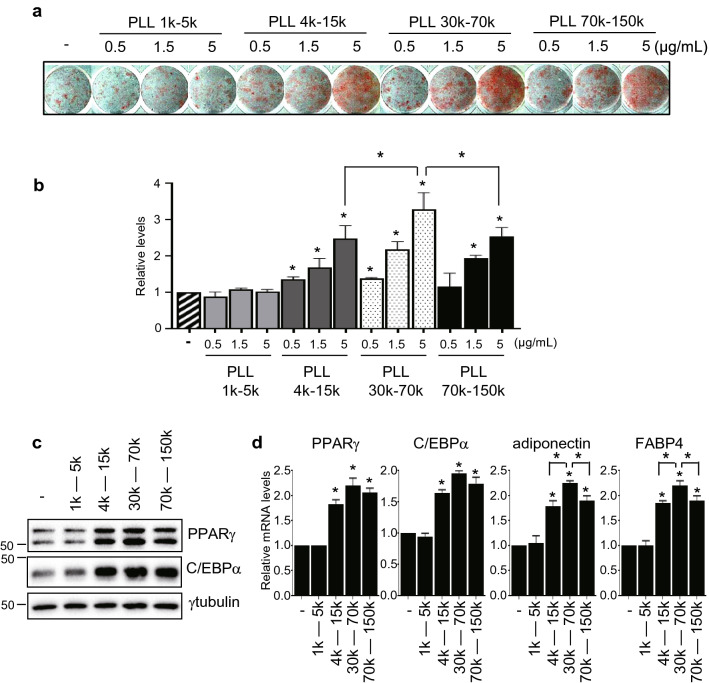
Poly-l-lysine (PLL) increases adipocyte differentiation. **a** Adipogenesis was induced as described in the supplementary information (Fig. S1). Briefly, 3T3-L1 cells were cultured in a differentiation-inducing medium containing different MW PLLs at indicated concentrations throughout differentiation. The cells were stained with Oil Red O on Day 8 after inducing differentiation. **b** The levels of Oil Red O staining were quantified. The stained levels of differentiated cells in the differentiation-inducing medium without PLL were set to 1, and the other values were calculated relative to this value (n = 4, average ± SEM). **c**, **d** Cells were differentiated using a differentiation-inducing medium containing different MW PLLs at a concentration of 5 μg/mL throughout differentiation. On Day 8 of adipogenesis, cell lysates (**c**) and total RNA (**d**) were harvested and subject to western blotting and real-time RT-PCR, respectively. **d** The mRNA levels of differentiated cells without PLL were set to 1, with other values calculated relative to this value (n = 3, average ± SEM). The significance was assessed using the one-way ANOVA followed by Tukey’s post hoc test. *P < 0.05

To investigate the effects of PLL on adipocyte differentiation, we determined lipid droplet formation and adipocyte-specific gene expression under different PLL concentrations. Throughout adipogenesis, 3T3-L1 cells were treated with different MWs and concentrations of PLL (Fig. S1). Since the formation and accumulation of lipid droplets represent the degree of adipocyte differentiation, we stained lipid droplets using Oil Red O to estimate adipocyte differentiation. While PLLs with a MW between 4 and 150 kDa increased lipid accumulation, those with a MW between 1 and 5 kDa did not make any difference even at high concentrations (Fig. 1a, b). PLLs with a MW between 30 and 70 kDa showed the maximum effect on lipid accumulation and the effect of PLL depends on its concentration (Fig. 1a, b). To confirm whether PLL-treated cells became adipocytes, the levels of the adipogenic marker genes PPARγ, C/EBPα, adiponectin, and FABP4 (fatty acid binding protein 4) were determined. The protein and mRNA levels of markers increased in PLL-induced 3T3-L1 cells (Fig. 1c, d) and corresponded to the amount of lipid in each sample. These results suggest that PLLs with a molecular weight greater than 4 kDa increase adipocyte differentiation and the effect of PLL on adipogenesis is dependent on its MW and concentration. To investigate the possibility that PLL has similar function in human cells, we monitored the extent of adipogenesis of primary human mesenchymal stem cells (hMSCs) incubated with PLL. The increase of lipid accumulation by PLLs with an MW greater than 4 kDa was also observed (Fig. S2). Unlike in 3T3-L1 cells, PLLs with an MW between 4 and 15 kDa showed the maximum effect on lipid accumulation and 1.5 µg/mL of PLLs was the most effective concentration for primary hMSC differentiation (Fig. S2). There are several types of polylysine and PDL has similar features with PLL so we checked the effect of PDL on 3T3-L1 adipogenesis. PDL also exhibited a similar MW- and concentration-dependent effect on adipogenesis with PLL (Fig. S3). Since the PLL precursor amino acid occurs naturally and the PDL precursor does not, our research focused on PLL.

#### Effects of PLLs are associated with insulin signaling and the cAMP signal pathway

**Fig. 2:**
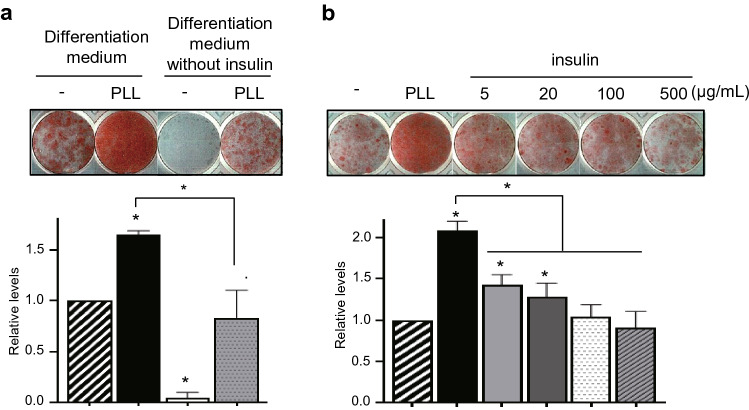
PLL acts as a substitute for insulin and enhances its effect on adipocyte differentiation. **a**, **b** The procedure for inducing adipogenesis is described in the supplementary information (Fig. S4, S5). **a** Cells were incubated with or without PLL (5 μg/mL, 30–70 kDa) in a differentiation-inducing medium with or without insulin throughout adipogenesis. On Day 8 of adipogenesis, the cells were stained with Oil Red O (upper panel). The levels of Oil Red O staining were quantified (lower panel). The stained levels of differentiated cells in the differentiation-inducing medium without PLL were set to 1, and the other values were calculated relative to this value (*n* = 3, average ± SEM). **b** Cells were incubated in a differentiation-inducing medium with PLL (5 μg/mL, 30–70 kDa) or insulin at the indicated concentration throughout adipocyte differentiation. On Day 8 of adipogenesis, cells were stained with Oil Red O (upper panel). The levels of Oil Red O staining were quantified (lower panel). The stained levels of differentiated cells in the differentiation-inducing medium without PLL were set to 1, and the other values were calculated relative to this value (*n* = 3, average ± SEM). The significance was evaluated using the one-way ANOVA followed by Tukey’s post hoc test. *P < 0.05

**Fig. 3:**
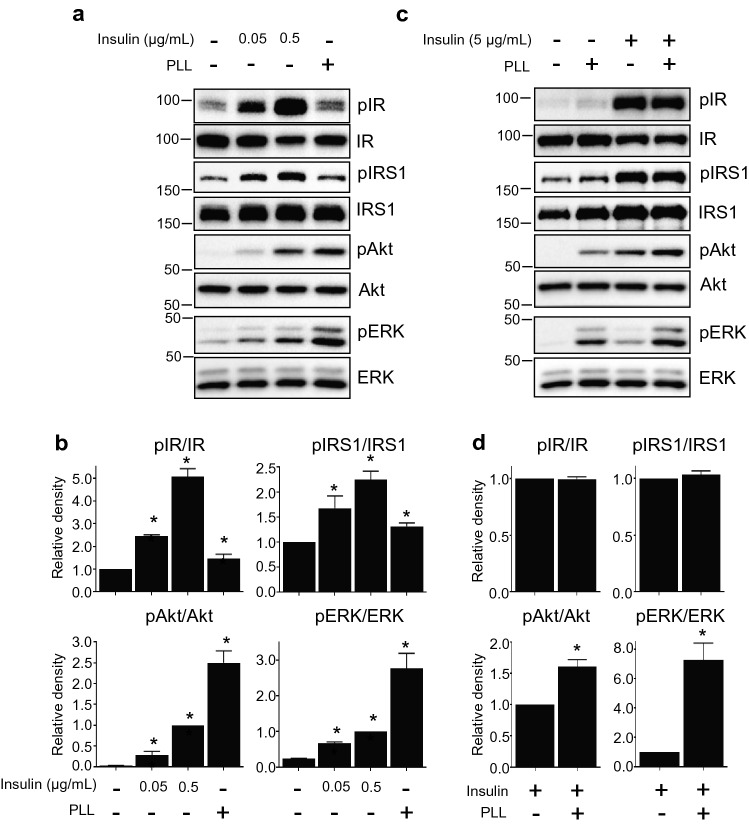
PLL stimulates insulin signaling. **a**, **c** Growth-arrested 3T3-L1 preadipocytes were treated with insulin at the indicated concentration or PLL 5 μg/mL (30–70 kDa) for 15 min. Protein samples were prepared for western blotting. **b, d** Each band of **a,** **c** was quantified. The band densities of non-treated cells were set to 1, and the other values were calculated relative to this value (*n* = 3, average ± SEM). The significance was assessed using the Mann–Whitney *U*-test. *P < 0.05 vs. the value of non-treated cells

To investigate the role of PLL in adipogenesis, we compared the effects of PLL with adipogenic inducers such as insulin, DEX, and IBMX, which are essential for 3T3-L1 differentiation. In the differentiation medium without insulin and PLL, adipogenesis was not initiated (Fig. 2a). Interestingly, when PLL was added to insulin-free differentiation medium, preadipocytes differentiated into adipocytes at similar levels of differentiation observed in insulin-contained differentiation medium, although the differentiation levels were lower than those of cells differentiated in the differentiation medium containing PLL and insulin (Fig. S4, Fig. 2a). To determine whether adding an extra amount of insulin along with PLL had a similar effect, 3T3-L1 preadipocytes were incubated in a differentiation medium with different concentrations of insulin during the entire period of adipogenesis (Fig S5). Although additional insulin increased lipid accumulation compared to the control, PLL still exhibited further enhancement of adipogenesis (Fig. 2b). These results suggest that PLL substituted and enhanced the effect of insulin on adipogenesis. Insulin signaling is the major regulatory pathway that controls adipogenesis and insulin stimulates this pathway (Smith et al. 1988). To investigate whether PLL mimicked or replaced the effect of insulin on adipogenesis, we monitored the status of proteins involved in the insulin signal pathway. Although insulin activates both IGF1 and insulin signaling during adipogenesis (Smith et al. 1988), PLL did not induce the phosphorylation of the IGF1 receptor (Fig. S6). Treatment with 5 µg/mL PLL for 15 min phosphorylated not only IR, the most upstream component of insulin signal pathway, but also IRS1, Akt, and ERK, downstream and representative signaling molecules in the pathway (Fig. 3a, b). These results suggest that PLL stimulates the insulin signal pathway during adipogenesis. The phosphorylation levels of upstream signal proteins such as IR and IRS1 showed a different pattern from that of downstream signal proteins such as Akt and ERK. In addition, the levels of pIR and pIRS1 increased by PLL were lower than those increased by 0.5 µg/mL insulin, while the levels of pAkt and pERK increased by PLL were higher than those increased by 0.5 µg/mL insulin (Fig. 3a, b). While the addition of PLL did not change the levels of IR and IRS1 phosphorylated by insulin alone, the levels of pAkt and pERK were increased by PLL (Fig. 3c, d). These results suggest a possibility that the insulin signaling pathway is not the only pathway for PLL to enhance adipogenesis.

**Fig. 4:**
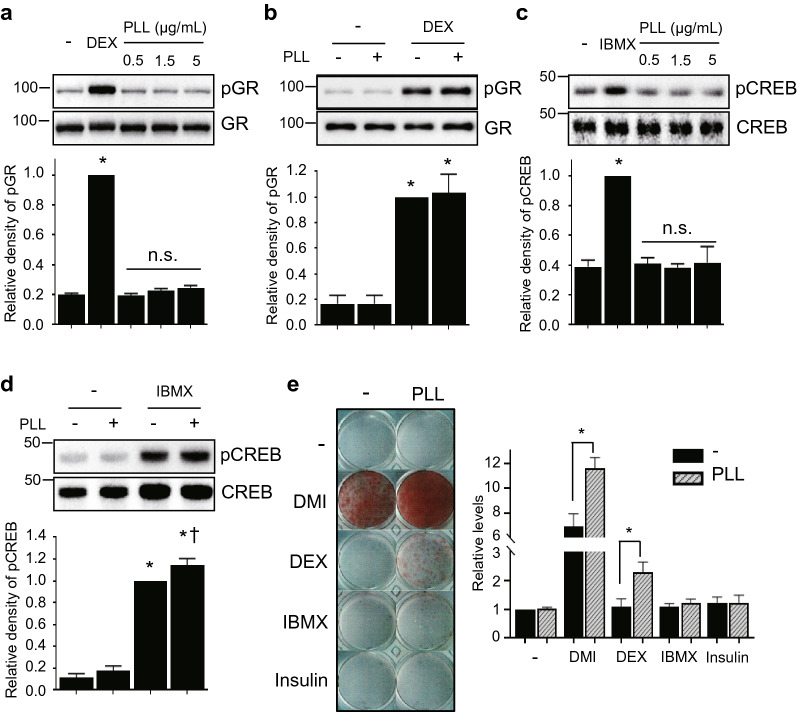
PLL exerts a positive effect on the insulin signaling pathway through IBMX but not through DEX. Growth-arrested 3T3-L1 preadipocytes were treated with DEX 0.25 μM, IBMX 0.5 mM, or PLL (30–70 kDa). **a** Cells were treated with DEX or PLL at the indicated concentration for 1 h. **b** Cells were treated with DEX or PLL 5 μg/mL for 1 h. **c** Cells were treated with IBMX or PLL at the indicated concentration for 30 min. **d** Cells were treated with IBMX or PLL 5 μg/mL for 30 min. **a–d** Protein samples were prepared for western blotting (upper panel). Each band was quantified (lower panel). The values of DEX or IBMX only-treated cells were set to 1, and the other values were calculated relative to this value (*n* = 3, average ± SEM). The significance was determined using the Mann–Whitney *U*-test. *P < 0.05 vs. the value of non-treated cells. ^†^*P* = 0.05 vs. the value of IBMX only-treated cells. *n.s*., not significant vs. the value of non-treated cells. **e** Adipogenesis was induced as shown in the supplementary information (Fig. S7). 3T3-L1 preadipocytes were incubated with or without PLL (5 µg/mL, 30–70 kDa) in the medium containing DEX (0.25 µM), IBMX (0.5 mM), or insulin (5 or 1 µg/mL). Cells were stained with Oil Red O on Day 8 of adipogenesis (left panel), and the levels of Oil Red O staining were quantified (right panel). The stained levels of non-induced differentiation and without PLL-treated cells were set to 1, and the other values were calculated relative to this value (n = 3, average ± SEM). The significance was calculated using the Mann–Whitney *U*-test. *P < 0.05

DEX and IBMX are also required to induce adipocyte differentiation of 3T3-L1. GRs are a major phosphorylation target of DEX, a synthetic GR ligand; however, GR phosphorylation was not affected by PLL treatment (Fig. 4a, b). IBMX, a phosphodiesterase inhibitor, increases cAMP levels and activates protein kinase A (PKA), whose activity is dependent on cAMP levels. Since CREB is phosphorylated by PKA, we compared the level of CREB phosphorylation after IBMX and PLL treatment. The levels of pCREB were not changed by PLL alone (Fig. 4c). However, PLL slightly increased CREB phosphorylation when preadipocytes were treated with IBMX (Fig. 4d), indicating that PLL can influence the cAMP signal pathway, although the effect was not statistically significant. Since PLL relates to the roles of insulin and IBMX, we determined whether a combination of PLL and DEX, IBMX, or insulin respectively induced adipocyte differentiation (Fig. S7). While the treatment with PLL/IBMX or PLL/insulin failed to induce lipid droplet formation, only the PLL/DEX combination induced adipogenesis (Fig. 4e), supporting the results of the signaling pathway analysis. Taken together, these results suggest that PLL positively affects adipocyte differentiation via several pathways, including insulin signaling and cAMP signaling.

#### Effect of PLL on adipocyte differentiation depends on the stage of differentiation

**Fig. 5:**
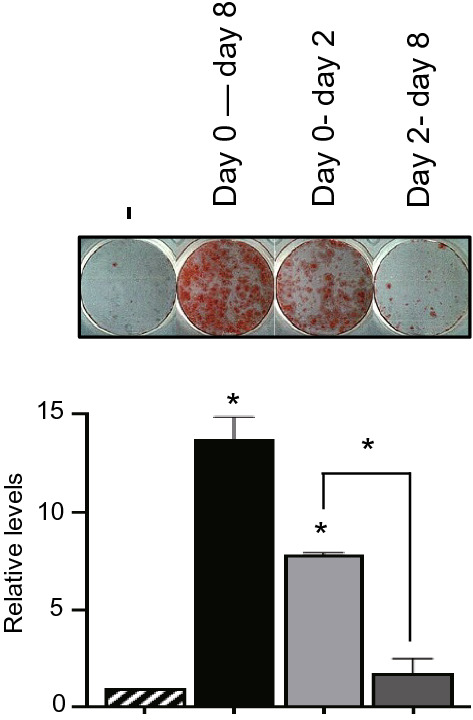
Days 0–2 after inducing differentiation are important for the effects of PLL on adipogenesis. Adipocyte differentiation was induced as shown in the supplementary information (Fig. S8). Briefly, cells were incubated in a differentiation-inducing medium with PLL (5 μg/mL, 30–70 kDa) during the indicated period. After 8 days, the cells were stained with Oil Red O (upper panel). The levels of Oil Red O staining were quantified (lower panel). The stained levels of incubated cells in differentiation-inducing medium without PLL were set to 1, and the other values were calculated relative to this value (*n* = 3, average ± SEM). The significance was determined using the one-way ANOVA followed by Tukey’s post hoc test. *P < 0.05

Preadipocytes differentiate into adipocytes via mitotic clonal expansion (MCE) and terminal differentiation stages (Guo et al. 2015). After the induction of 3T3-L1 differentiation, MCE begins and continues for 48 h, followed by terminal differentiation. To determine the adipogenesis stage affected by PLL, 3T3-L1 cells at different developmental stages were incubated with PLL: the entire period of adipogenesis, from Day 0–2 after inducing differentiation, and from Day 2–8 after inducing differentiation (Fig. S8). Incubation with PLL during the entire period of adipogenesis exhibited an apparent increase in adipocyte numbers and incubation with PLL on Day 2 after inducing differentiation also led to a moderate increase. However, adipogenesis was not enhanced by PLL when the cells were treated with PLL from Day 2 after inducing differentiation (Fig. 5). Since the adipogenic gene expression program started during several rounds of MCE (Tang et al. 2003), the increase of cell number is closely correlated with adipocyte differentiation. The number of cells incubated in the differentiation-inducing medium with PLL increased more than the number of cells incubated in the differentiation-inducing medium without PLL (Fig. S9), and the increased number of cells was maintained (Fig. S9). These results suggest that the effects of PLL on adipocyte differentiation depend on the stage of adipogenesis, particularly the MCE stage and the increase of cell proliferation by PLL may contribute to the enhancement of adipogenesis.

## Discussion

3T3-L1 preadipocytes have been adopted as a model cell line for studying adipocyte biology, and have been used to elucidate many characteristics and regulatory mechanisms of adipogenesis. 3T3-L1 adipogenesis can be promoted via the sequential addition of different culture media or the addition of peptide hormones. Glucagon-like peptide (GLP-1), a gut-produced peptide, has been reported to play an important role in the metabolism and regulation of 3T3-L1 adipogenesis via the Akt signaling pathway (Yang et al. 2013). The gastric inhibitory polypeptide (a glucose-dependent insulinotropic polypeptide) also activates Akt signaling, leading to enhanced adipocyte development (Song et al. 2007). These peptide hormones are secreted naturally and regulate adipogenesis in an insulin-mediated manner. Although PLL is not a hormone, we found that it could enhance adipogenesis and even replace the role of insulin as an adipogenic inducer. Our study also revealed that the activation of insulin signaling is involved in the regulatory mechanisms of PLL during adipogenesis.

In this study, we determined the function of PLLs as an adipogenic inducer in 3T3-L1 cells. This effect of PLLs was also observed in hMSCs but differentiation media of hMSC is complex so that it was difficult to find out the in vivo function of PLL (Fig. S2). While PLL replaces insulin as an adipogenic inducer of 3T3-L1, PLL may only accelerate cell proliferation or affect cell attachment of hMSC. Interestingly, incubation with PDL exhibited a similar MW- and concentration-dependent effect on adipogenesis with PLL (Fig. S3). The similar effect of PLL and PDL may suggest the polycations and chain length of two polylysines may contribute to 3T3-L1 adipogenesis and their chirality does not exert effect.

**Fig. 6:**
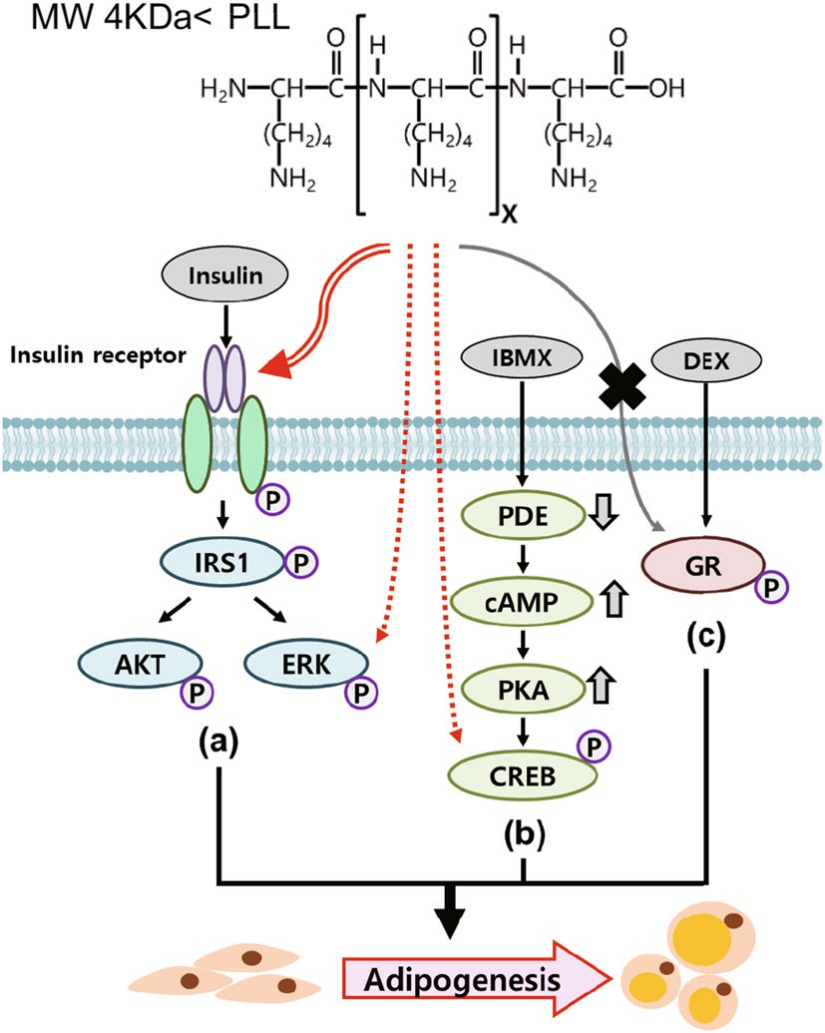
A proposed mechanism of signaling pathways that PLL activates in 3T3-L1 adipogenesis. Three essential adipogenic components such as insulin, IBMX, and DEX activate insulin signaling pathway (**a**), cAMP signaling pathway (**b**), and glucocorticoid receptor signaling pathway (**c**) respectively. PLL phosphorylates IR and also activates ERK and AKT indirectly. PLL increases phosphorylation of CREB, indicating the involvement of cAMP pathway. Dotted lines indicate an ambiguous mechanism that was influenced by PLL

Since PLL is a positively charged polymer, it can be used as a drug delivery vehicle by forming soluble components with negatively charged drugs and as a cell culture additive to improve cell adherence to cultureware. In this study, we investigated the effects of synthetic PLL homopolymers on 3T3-L1 adipogenesis and the underlying differentiation regulatory mechanisms. Several rounds of MCE are important for adipocyte differentiation because the adipogenic gene expression program started during this period. We showed that MCE is the main period of adipogenesis influenced by PLLs (Fig. 5). And we also observed the further increase of cell number during MCE and the maintenance of increased cell number in incubation with PLLs (Fig. S9).To induce adipogenesis, 3T3-L1 preadipocytes should be stimulated by a combination of insulin, DEX, and IBMX (Fig. 6). We compared the effects of PLL on adipogenesis with those of the three adipogenic inducers, insulin, DEX, and IBMX to elucidate the role of PLL in adipogenesis. Each adipogenic inducer contributes to adipogenesis by activating different signaling pathways. We found that PLL has similar functions as insulin, since it induces adipocyte differentiation in insulin-free conditions (Fig. 2a) and activates the insulin signaling pathway (Figs. 3, 6a), although PLL does not have a function as a trigger for IGF1 signaling (Fig. S6). The activation of insulin signaling by PLL is consistent with the results of previous studies that PLL activates insulin receptor protein kinase by interacting with its β-subunit (Fujita-Yamaguchi et al. 1989; Rosen and Lebwohl 1988). Furthermore, PLL increased the levels of pAkt and pERK compared to treatment with insulin alone when preadipocytes were treated with insulin and PLL at the same time (Fig. 3c, d), indicating the enhancement of the insulin signaling pathway. Interestingly, PLL strongly stimulated the phosphorylation of ERK than insulin (Fig. 3) and further increased the levels of pAkt and pERK compared to those of pIR and pIRS1 (Fig. 3a, b). In addition, the degree of adipocyte differentiation after treatment with PLL and insulin was higher than that after treatment with insulin alone, although insulin was used at a higher concentration and for a longer incubation time (Fig. 2b). These results suggest that PLL may affect another pathway that stimulates adipocyte differentiation (dotted line in Fig. 6a). PLLs did not phosphorylate GR, which can be activated by DEX, indicating that the effects of PLL on adipogenesis were not related to DEX (Fig. 6c). We did not observe the phosphorylation of CREB by PLL alone (Fig. 4c), although CREB can be phosphorylated by ERK activation as a PKA-independent pathway (Petersen et al. 2008). However, the addition of PLL with IBMX in the culture media showed an increase in CREB phosphorylation, which is dependent on cAMP (Fig. 4d), indicating the possibility of a positive role of PLL on the cAMP signal pathway (dotted line in Fig. 6b). While treatment with DEX, IBMX, or insulin alone failed to differentiate preadipocytes, the combined treatment of PLL and only DEX, and not IBMX or insulin, induced adipogenesis (Fig. 4e), indicating that PLL is mainly involved in the DEX-independent pathway. Taken together, these results suggest that PLL may complement the role of insulin as well as function as a mimetic or replace insulin to differentiate preadipocytes. Further studies are required to elucidate the regulatory mechanism that PLL affects adipocyte differentiation.

Recently, PLL has been developed as a useful component in various biological experiments and biomedical applications. The function of PLL of activating the IR signaling pathway enables to extend its application but using PLL as a drug carrier and for immunization purposes require a careful interpretation of its use and further investigation. Since PLL is biologically active and IR signaling initiates cascades that result in different biological effects such as energy metabolism, cellular growth, proliferation, and development (Denley et al. 2005), the use of PLL as a carrier or adjuvant may cause overestimation or distortion of the effect of the original molecules involved in the process.

In summary, PLLs with an MW greater than 4 kDa enhanced adipogenesis and the activation of insulin signaling was significantly involved to produce this effect. However, treatment with a high concentration of insulin could not exert a similar effect on differentiation compared to that of PLL and the levels of pAkt and pERK increased by PLL were higher than those of other signaling molecules, pIR and pIRS1, indicating that insulin signaling is one of the regulatory mechanisms in which PLL is involved. We found that PLL also affects the cAMP signal pathway, although to a lesser extent. In addition, MCE is an important stage of adipogenesis for inducing the effects of PLL since adipocyte differentiation hardly increased when the cells were treated with PLL after the MCE stage. In conclusion, we identified novel effects of PLL on adipocyte differentiation and suggested that PLL could act as a substitute and a complement for insulin in adipocyte differentiation. Further studies on the regulatory mechanisms of PLL will not only enhance our knowledge of the action of PLL but also lead to the development of new applications.

## Supplementary Information

Below is the link to the electronic supplementary material.

Supplementary file1 (TIF 184 KB)

Supplementary file2 (TIF 183 KB)

Supplementary file3 (TIF 41 KB)

Supplementary file4 (TIF 46 KB)

Supplementary file5 (TIF 46 KB)

Supplementary file6 (TIF 77 KB)

Supplementary file7 (TIF 36 KB)

Supplementary file8 (TIF 51 KB)

Supplementary file9 (TIF 43 KB)

## Data Availability

The submitted work is original and is not published elsewhere in any form or language.
