# Supplementary figures and images for: α-Poly-l-lysine functions as an adipogenic inducer in 3T3-L1 preadipocytes
Source: Amino Acids. 2021 Mar 20;53(4):587–96. doi: 10.1007/s00726-020-02932-2 (PMC8107076; doi:10.1007/s00726-020-02932-2)

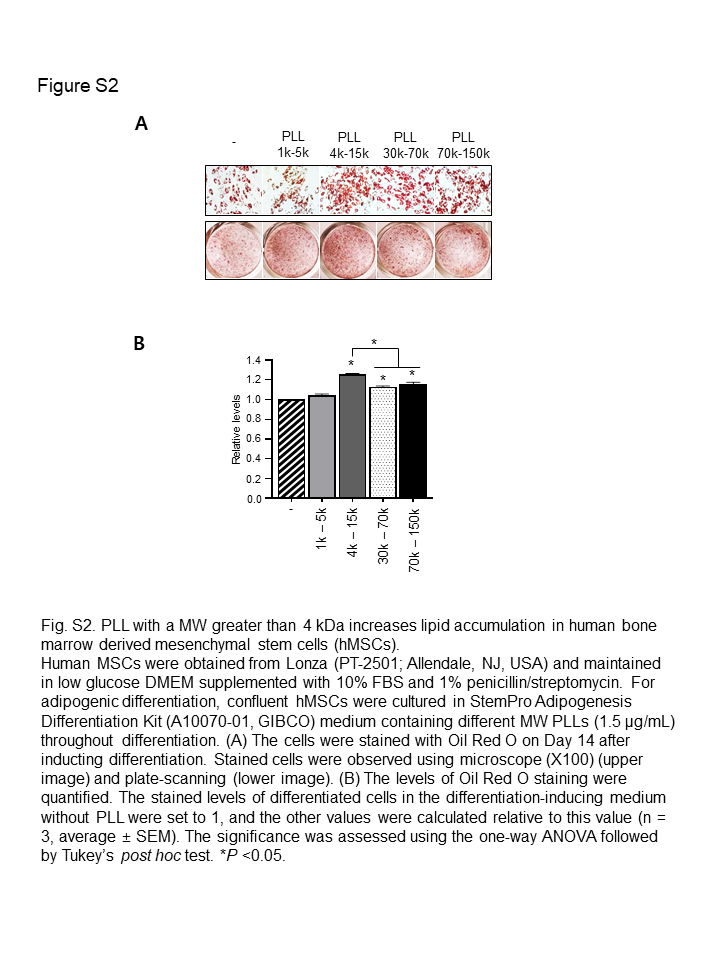

Supplement: Supplementary file 1 — Supplementary file1 (TIF 184 KB) [file 726_2020_2932_MOESM1_ESM.tif]

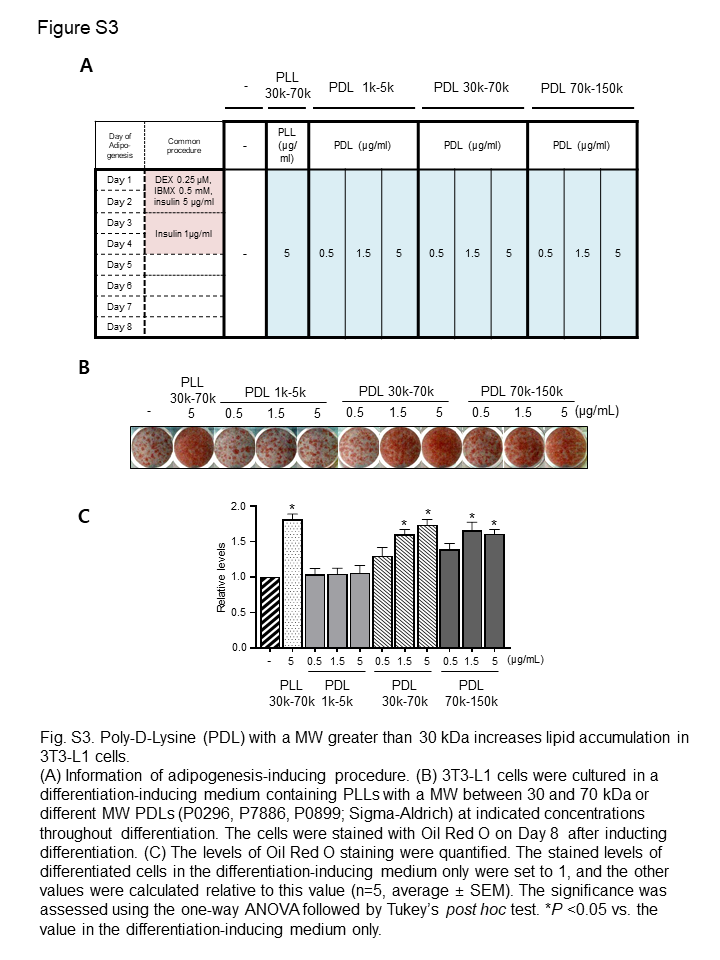

Supplement: Supplementary file 2 — Supplementary file2 (TIF 183 KB) [file 726_2020_2932_MOESM2_ESM.tif]

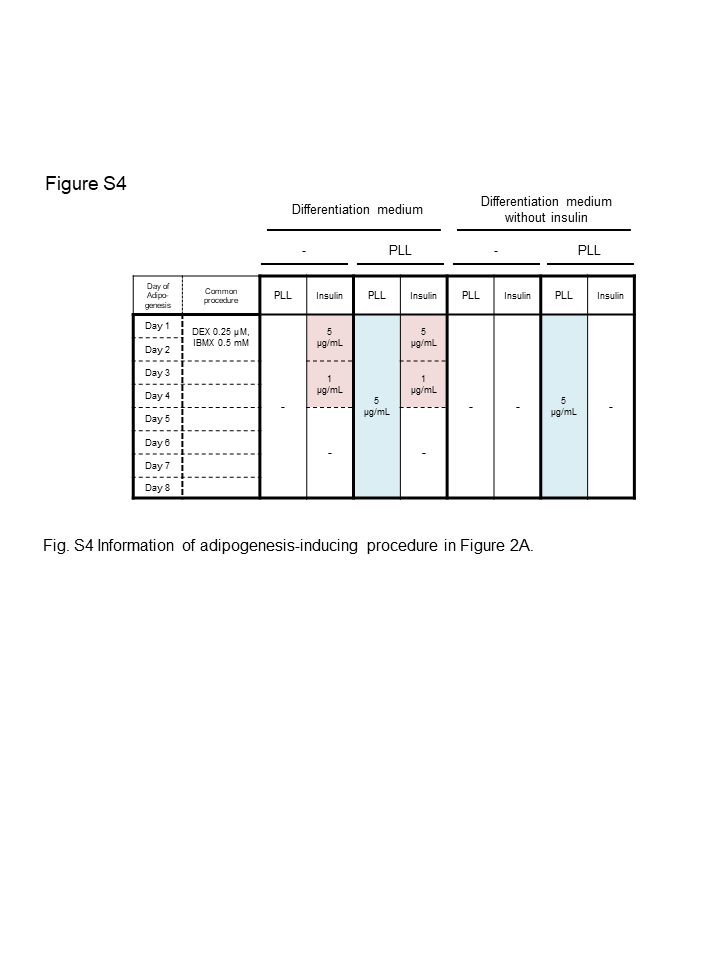

Supplement: Supplementary file 3 — Supplementary file3 (TIF 41 KB) [file 726_2020_2932_MOESM3_ESM.tif]

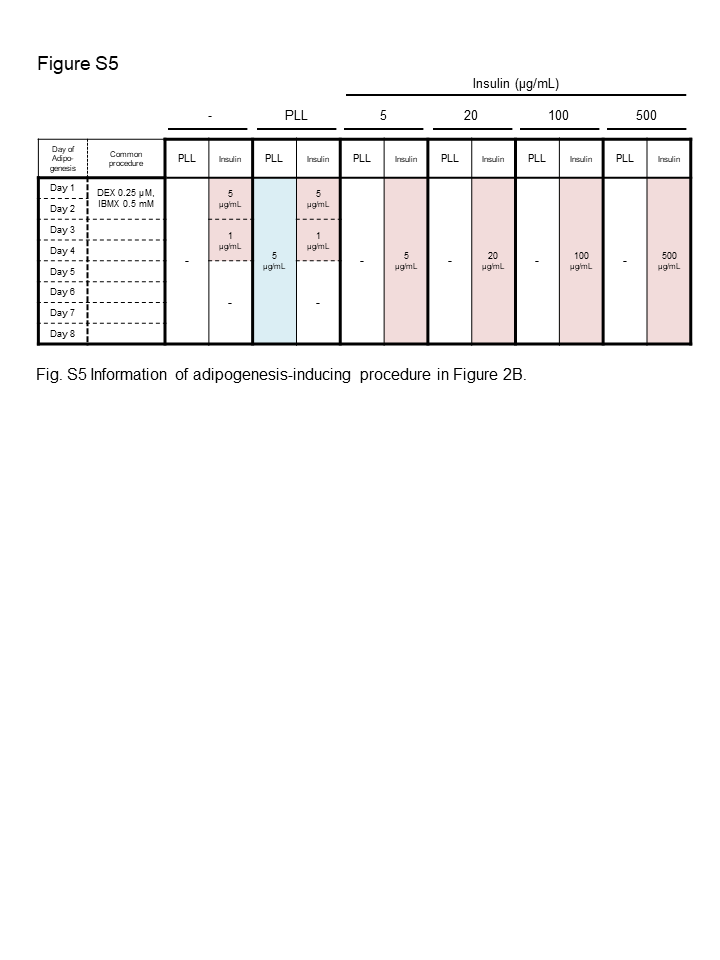

Supplement: Supplementary file 4 — Supplementary file4 (TIF 46 KB) [file 726_2020_2932_MOESM4_ESM.tif]

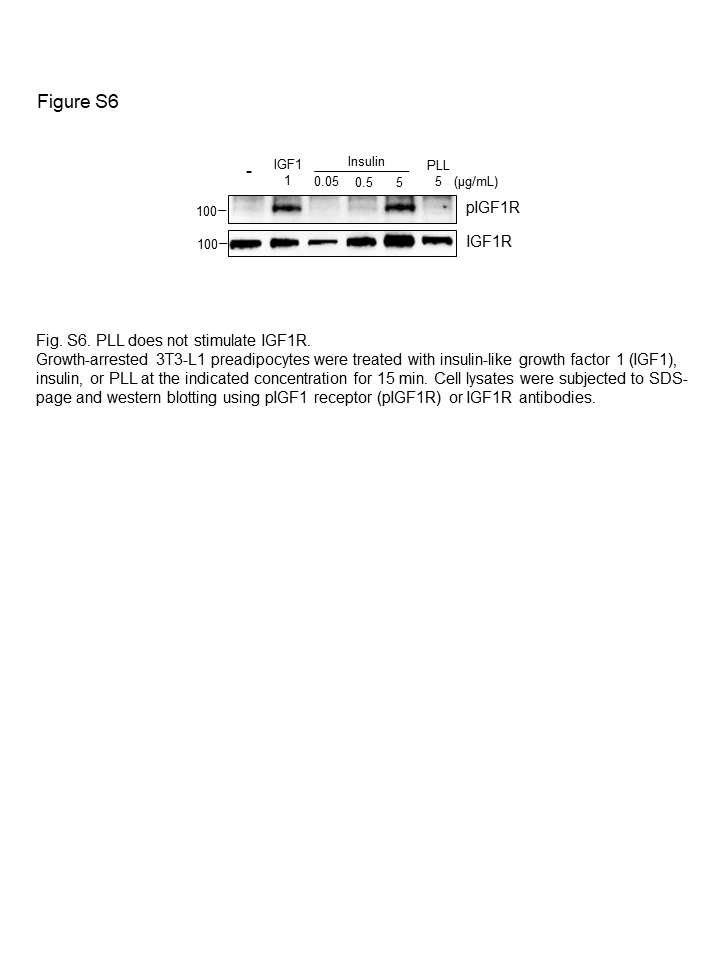

Supplement: Supplementary file 5 — Supplementary file5 (TIF 46 KB) [file 726_2020_2932_MOESM5_ESM.tif]

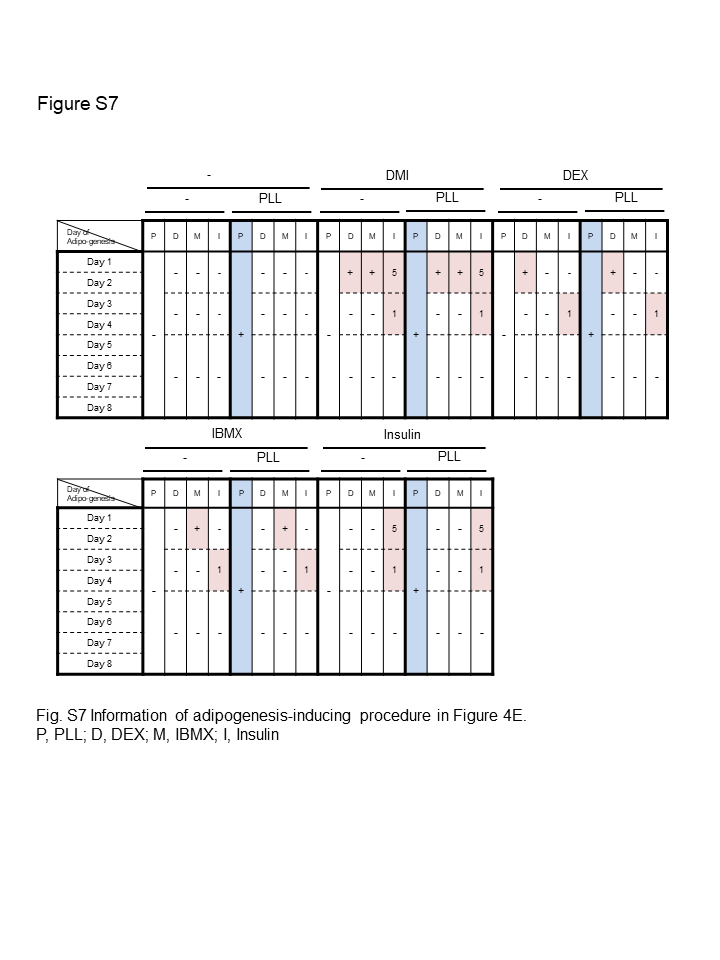

Supplement: Supplementary file 6 — Supplementary file6 (TIF 77 KB) [file 726_2020_2932_MOESM6_ESM.tif]

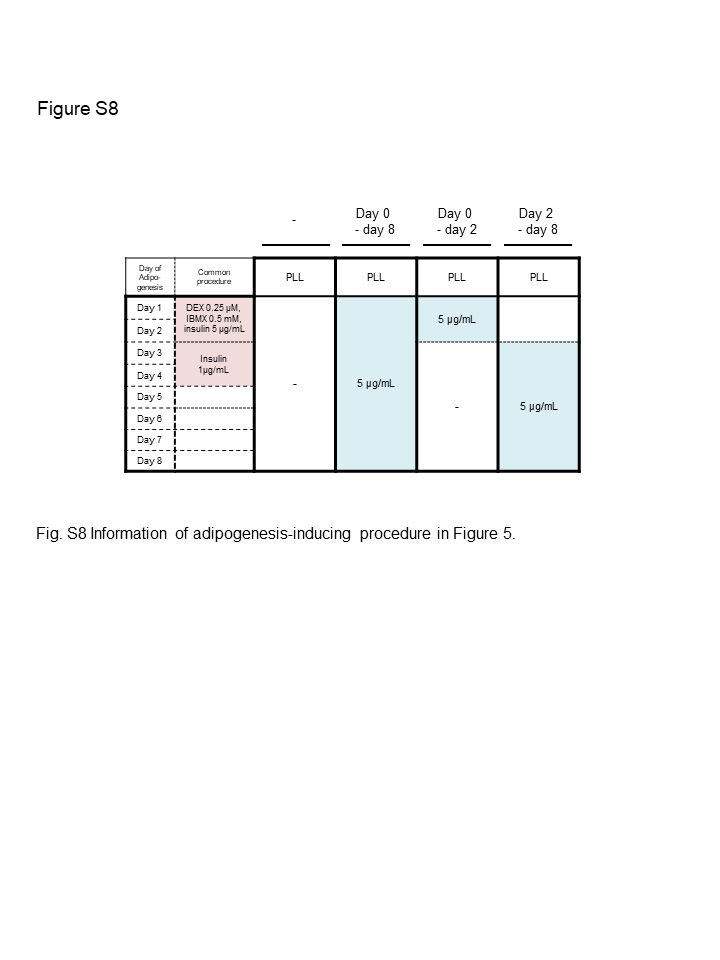

Supplement: Supplementary file 7 — Supplementary file7 (TIF 36 KB) [file 726_2020_2932_MOESM7_ESM.tif]

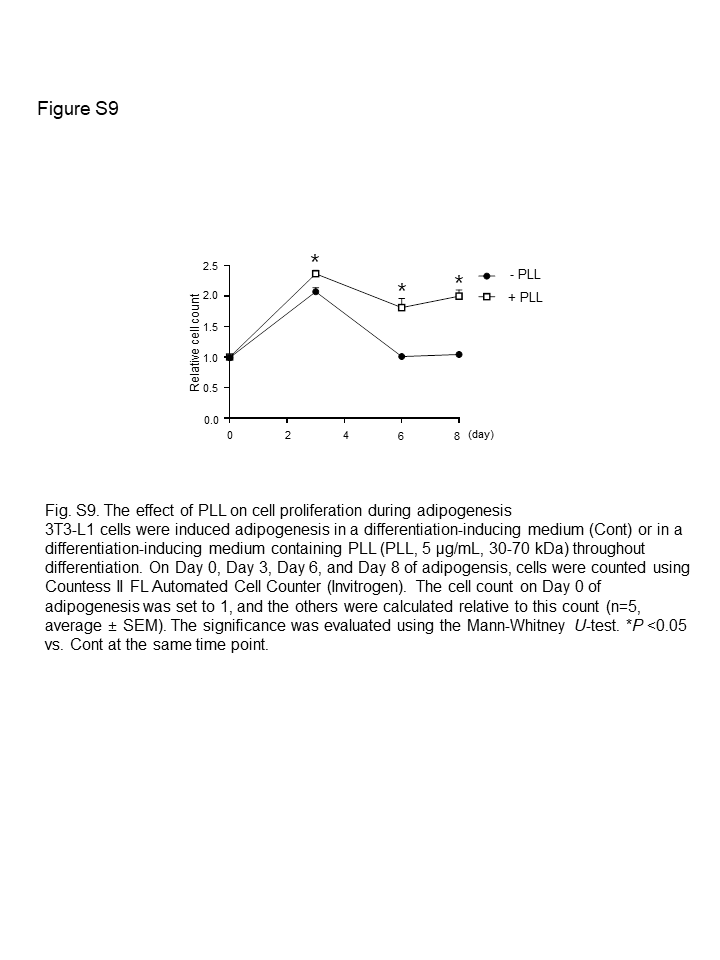

Supplement: Supplementary file 8 — Supplementary file8 (TIF 51 KB) [file 726_2020_2932_MOESM8_ESM.tif]

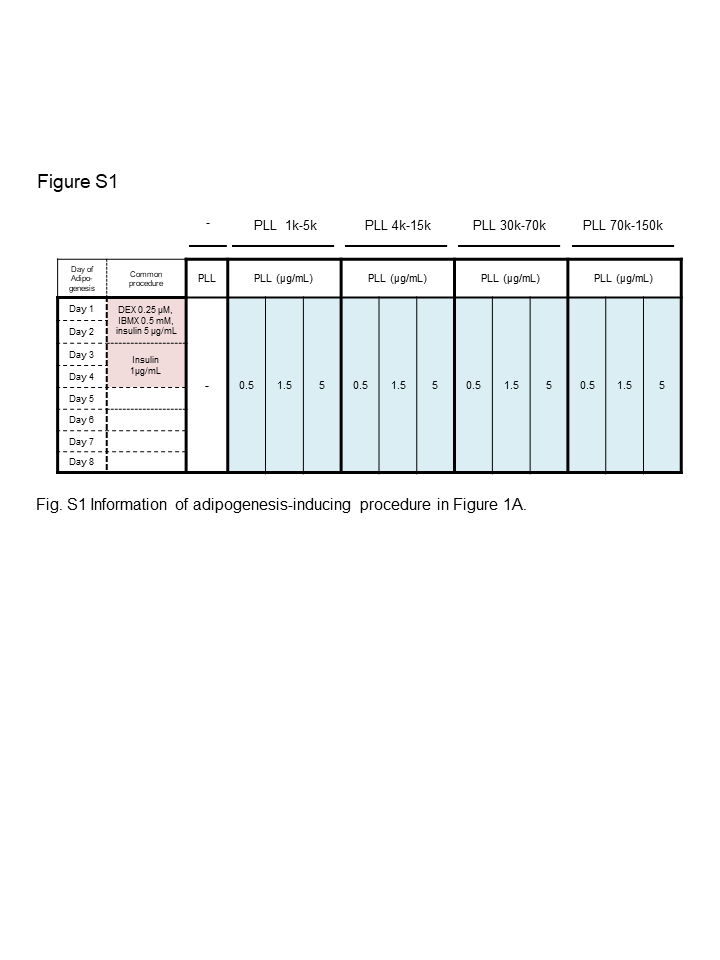

Supplement: Supplementary file 9 — Supplementary file9 (TIF 43 KB) [file 726_2020_2932_MOESM9_ESM.tif]
